# Effects of CoQ10 Supplementation on Lipid Profiles and Glycemic Control in Patients with Type 2 Diabetes: a randomized, double blind, placebo-controlled trial

**DOI:** 10.1186/s40200-014-0081-6

**Published:** 2014-07-25

**Authors:** Hoda Zahedi, Shahryar Eghtesadi, Soroush Seifirad, Neshat Rezaee, Farzad Shidfar, Iraj Heydari, Banafsheh Golestan, Shima Jazayeri

**Affiliations:** 1 Obesity and Eating Habits Research Center, Endocrinology and Metabolism Molecular -Cellular Sciences Institute, Tehran University of Medical Sciences, Tehran, Iran; 2 Department of Nutrition, School of Public Health, Iran University of Medical Sciences, Tehran, Iran; 3 Endocrinology and Metabolism Research Center, Endocrinology and Metabolism Clinical Sciences Institute, Tehran University of Medical Sciences, Tehran, Iran; 4 Institute of Endocrinology and Metabolism, Firouzgar Hospital, Iran University of Medical Sciences, Tehran, Iran; 5 Department of Epidemiology and Biostatistics, School of Public Health, Tehran University of Medical Sciences, Tehran, Iran

**Keywords:** Coenzyme Q10, Diabetes mellitus type 2, Blood glucose, Oxidative stress, Inflammation

## Abstract

**Background:**

Low grade inflammation and oxidative stress are the key factors in the pathogenesis and development of diabetes and its complications. Coenzyme Q10 (CoQ10) is known as an antioxidant and has a vital role in generation of cellular energy providing. This study was undertaken to evaluate the effects of CoQ10 supplementation on lipid profiles and glycemic controls in patients with diabetes.

**Methods:**

Fifty patients with diabetes were randomly allocated into two groups to receive either 150 mg CoQ10 or placebo daily for 12 weeks. Before and after supplementation, fasting venous blood samples were collected and lipid profiles containing triglyceride, total cholesterol, low-density lipoprotein cholesterol (LDL-C) and high-density lipoprotein cholesterol (HDL-C) and glycemic indices comprising of fasting plasma glucose (FPG), insulin and hemoglobin A_1_C (HbA_1_C) were measured. Insulin resistance was calculated using HOMA-IR index.

**Results:**

Forty patients completed the study. After intervention FPG and HbA_1_C were significantly lower in the CoQ10 group compared to the placebo group, but there were no significant differences in serum insulin and HOMA-IR between the two groups. Although total cholesterol did not change in the Q10 group after supplementation, triglyceride and HDL-C significantly decreased and LDL-C significantly increased in the CoQ10 group.

**Conclusion:**

The present study showed that treatment with Q10 may improve glycemic control with no favorable effects on lipid profiles in type 2 patients with diabetes.

**Trial registration:**

IRCT registry number: IRCT138806102394N1

## Introduction

Beyond certain medical treatments, food diets and supplementation plays a key role in the management of chronic diseases and promoting health status of human beings in the 21th century [1]. Previous studies have shown that the dysfunction of endothelium caused by oxidative stress [2] and inflammation [3] resulted from hyperglycemia is the key factor in the incidence and development of vascular complications of diabetes; Hence antioxidants intake is an appropriate intervention to improve this condition [4].

Coenzyme Q10 (CoQ10), a kind of benzoquinone, also known as ubiquinone, is a vitamin like antioxidant that can be synthesized in the body and is found in all biologic membranes [5],[6]. Also it is a vital component of the mitochondrial electron transport chain that has an intermittent role in ATP production [2]. Moreover, this ubiquinone may cause regeneration of reduced forms of vitamin E and C [4] and it shows stronger antioxidant properties than vitamin E [2]. In addition, CoQ10 can protect the lipids of membranes and blood circulation against peroxidation [7].

Research investment toward discovering appropriate supplements in complementary medicine has a high importance for optimal management of patient with diabetes [8]. Studies have shown that amongst the clinical supported supplements, Q10 supplements are one of the most popular ones that might be valuable in the management of neurodegenerative and cardiovascular diseases [5],[7],[9]-[11].

The importance of this supplementation in inherited diabetes is well known [12]-[14]. It has been shown that the serum concentrations of Coenzyme Q10 are reduced in patient with diabetes [15]. Regarding the benefits of Q10 supplementation shown in animal studies [3],[16]-[18] and controversial findings of human trials [4],[19]-[21], this study was conducted to evaluate the effects of Q10 supplementation on lipid profiles and glycemic control in type 2 patients with diabetes.

## Methods and material

In a double blind randomized controlled trial, fifty patients with diabetes, aged less than 75 years were randomly allocated into two groups. They received either 150 mg CoQ10 supplement (Natural Factors Inc. Canada) or 150 mg placebo (maize starch) in three divided 50 mg doses daily for 12 weeks. The inclusion criteria were as follows: diagnosis of type 2 diabetes which were defined as fasting plasma glucose (FPG) ≥126 mg/dl or 2 hours plasma glucose (2HPG) ≥200 mg/dl), having diabetes for at least one year, Hemoglobin A1C (HbA1C) <9%, serum concentration of triglyceride less than 400 mg/dl. Exclusion criteria were body mass index (BMI) more than 40 Kg/m^2^, history of myocardial infarction or stroke, insulin therapy, incidence of diseases (such as liver, renal or thyroid disorders), consumption of antioxidant supplements in the past two months, Warfarin intake, uncontrolled hypertension and pregnancy or lactation. The patients who consumed less than 70% of supplements were also excluded from the study.

Venous blood samples (10 ml) were collected between 8–9 A.M. after fasting for 10–12 hours at baseline and after 12 weeks of supplementation with CoQ10. The measurements were performed on frozen serum samples. The serum levels of Q10 were measured before and after intervention using high-pressure liquid chromatography (HPLC) method and an Immunodiagnostik kit (Immunodiagnostik/Bensheim, Germany). The serum levels of triglyceride were measured using GOP-PAP method and a Pars Azmoon kit (Pars Azmoon Inc.,Tehran, Iran). Total cholesterol was tested using the same kit through Liasys auto analyzer device (Liasys, Roma, Italy). Serum high density lipoprotein cholesterol (HDL-C) levels also were determined using sedimentary method with the same kit. Low density lipoprotein cholesterol (LDL-C) levels were calculated indirectly using Friedewald Equation:

(1) LDL.C=T.C‐HDL.C‐TG/5

Glycemic control indices included FPG, homeostatic model assessment (HOMA-IR) and HbA_1_C. FPG was measured using glucose oxidase method (Pars Azmoon Inc., Tehran, Iran). Insulin levels were measured using RIA method and Immonotech kit. HOMA-IR was calculated using following formula:

(2) $$\mathrm{HOMA}-\mathrm{IR}=\frac{\mathrm{Insulin}\left(\mathrm{\mu m}/\mathrm{ml}\right)\times \mathrm{Glu}\cos e\left(\mathrm{mg}/\mathrm{dl}\right)}{405}$$

HbA_1_C was determined using chromatography method by DS5 Drew Scientific machine (ion exchange chromatography).

Dietary intakes were assessed using 24-hour dietary recall questionnaires before and after intervention. Energy and nutrient intakes were calculated by Nutritionist‑IV software (First Data Bank, The Hearst Corporation, San Bruno, CA).

The statistical tests were conducted by paired sample t-test, independent sample t-test and ANCOVA using the statistical package for the social sciences (SPSS), version 15; (SPSS; Inc., Chicago, IL, USA). Kolmogorov–Smirnov test was used to evaluate normality. Due to normal distribution pattern of our data, parametric studies were applied for statistical analysis. Data were presented as means and standard deviations. P < 0.05 was considered as statistically significant. Ethical approval was obtained from the Medical Ethics Committee of Iran University of Medical Sciences and the participants signed the informed consent.

## Results

Forty patients completed the 12 weeks study protocol. Ten patients were excluded from the study. Four patients lost to follow up and six patients consumed less than 70% of the given supplements.

Baseline characteristics and nutrients intakes of participants are shown in Tables 1 and 2. No statistically significant differences were observed between the two groups with regard to characteristics and nutrients intakes at baseline.

**Table 1:** Baseline characteristics of participants

**Variables**		**Q**_**10**_**(n = 20)**	**Placeo (n = 20)**	**P-value**
Age (years)		53.5 ± 9.7	58.8 ± 9.6	NS*
Diabetes duration (years)		7.4 ± 5.2	7.9 ± 6.7	NS
Sex	Female	9	12	NS
	Male	11	8	
Smoking	Yes	2	2	NS
	No	18	18	
Family history diabetes hypertension cardiovascular				
	Yes	14	9	NS
	No	6	11	
	Yes	13	10	NS
	No	7	10	
	Yes	7	10	NS
	No	13	10	
Kind of drug used	Hypoglycemic	4	4	NS
	Hypolipidemic	7	7	
	Antihypertensive	1	1	
	Combined	8	8	
Serum levels of Q10 (nmol/L)		1.44 ± 0.15	1.40 ± 0.18	NS

*NS: Not Significant.

P < 0.05 is considered as significant.

**Table 2:** Nutrient intake based on 24 hour dietary recall

**Variables**	**Before intervention (Mean ± SD)**	**After intervention (Mean ± SD)**	**P-value**
**Energy (Kcal)**			
Q10 (n = 20)	1466.3 ± 626.3	1505.8 ± 319.8	NS^(c)^
Placebo (n = 20)	1461.3 ± 407.1	1443.1 ± 340.0	NS
P-value	NS^*(a)^	NS^(b)^	
**Carbohydrate (g)**			
Q10 (n = 20)	220.6 ± 119.7	214.9 ± 59.4	NS
Placebo (n = 20)	212.2 ± 68.0	223.9 ± 78.0	NS
P-value	NS	NS	
**Protein (g)**			
Q10 (n = 20)	92.8 ± 127.8	86.1 ± 27.9	NS
Placebo (n = 20)	68.0 ± 36.0	58.3 ± 19.7	NS
P-value	NS	NS	
**Fat (g)**			
Q10 (n = 20)	78.5 ± 18.1	41.6 ± 19.5	NS
Placebo (n = 20)	39.1 ± 22.1	39.1 ± 13.8	NS
P-value	NS	NS	
**Fiber (g)**			
Q10 (n = 20)	13.1 ± 6.3	14.6 ± 2.8	NS
Placebo (n = 20)	13.1 ± 7.0	14.5 ± 10.1	NS
P-value	NS	NS	
**Cholesterol (mg)**			
Q10 (n = 20)	305.9 ± 750.3	149.8 ± 92.2	NS^(c)^
Placebo (n = 20)	328.1 ± 504.1	134.7 ± 59.9	NS
P-value	NS^*(a)^	NS^(b)^	
**Vitamin E (mg)**			
Q10 (n = 20)	73.3 ± 58.6	103.9 ± 123.9	NS
Placebo (n = 20)	75.1 ± 52.5	103.1 ± 147.0	NS
P-value	NS	NS	
**Vitamin C (mg)**			
Q10 (n = 20)	4.6 ± 3.1	4.3 ± 3.1	NS
Placebo (n = 20)	2.9 ± 1.9	2.7 ± 1.9	NS
P-value	NS	NS	
**Vitamin A (RE)**			
Q10 (n = 20)	718.3 ± 1170.8	685.7 ± 1407.6	NS
Placebo (n = 20)	2687.2 ± 7787.3	557.5 ± 525.8	NS
P-value	NS	NS	
**Riboflavin (mg)**			
Q10 (n = 20)	2.8 ± 7.1	1.2 ± 0.6	NS
Placebo (n = 20)	2.3 ± 4.5	1.3 ± 0.6	NS
P-value	NS	NS	
**Variables**	**Before intervention (Mean ± SD)**	**After intervention (Mean ± SD)**	**P-value**
**Zinc (mg)**			
Q10 (n = 20)	11.8 ± 24.1	6.3 ± 2.6	NS^(c)^
Placebo (n = 20)	7.9 ± 6.5	5.8 ± 2.5	NS
P-value	NS^*(a)^	NS^(b)^	
**Selenium (mg)**			
Q10 (n = 20)	0.09 ± 0.07	0.07 ± 0.04	NS
Placebo (n = 20)	0.08 ± 0.04	0.08 ± 0.04	NS
P-value	NS	NS	

*NS: Not Significant.

P < 0.05 is considered as significant.

^(a)^Based on independent t-test.

^(b)^Based on ANCOVA adjusted for baseline values.

^(c)^Based on paired t-test.

The serum levels of Q10 had no statistical significant differences between two groups before intervention but these levels were significantly higher in Q10 group (P < 0.05) after 12 weeks without significant change in the placebo group (P > 0.05).

There were no significant differences in the baseline values of lipid profile variables between the two groups (Table 3). Serum levels of triglyceride and HDL-C had no significant differences between the two groups at the end of the study, but LDL-C and total cholesterol were significantly higher in the Q10 group compared to the placebo group. Although LDL-C significantly increased after 12 weeks of supplementation with Q10, triglyceride and HDL-C decreased after intervention.

**Table 3:** Lipid profile indices before and after intervention

**Variable**	**Before intervention (Mean ± SD)**	**After intervention (Mean ± SD)**	**P-value**
**Triglyceride (mg/dl)**			
Q10 (n = 20)	136.90 ± 53.1	113.10 ± 50.3	0.004^(c)^
Placebo (n = 20)	146.25 ± 65.5	139.85 ± 64.9	NS
P-value	NS^*(a)^	NS^(b)^	
**Total cholesterol (mg/dl)**			
Q10 (n = 20)	143.05 ± 16.4	151.15 ± 24.8	NS
Placebo (n = 20)	145.90 ± 23.5	138.15 ± 23.3	NS
P-value	NS	0.02	
**LDL-C (mg/dl)**			
Q10 (n = 20)	74.55 ± 15.3	90.65 ± 18.7	0.001
Placebo (n = 20)	76.40 ± 21.6	71.15 ± 20.3	NS
P-value	NS	<0.001	
**HDL-C (mg/dl)**			
Q10 (n = 20)	42.40 ± 8.7	37.15 ± 10.7	0.002
Placebo (n = 20)	40.25 ± 10.5	38.85 ± 10.4	NS
P-value	NS	NS	

*NS: Not Significant.

P < 0.05 is considered as significant.

^(a)^Based on independent t-test.

^(b)^Based on ANCOVA adjusted for baseline values.

^(c)^Based on paired t-test.

CoQ10: Coenzyme Q10; LDL-C: Low-Density Lipoprotein Cholesterol; HDL: High-Density Lipoprotein Cholesterol.

The results of glycemic control are shown in Table 4. After intervention FPG and HbA_1_C were significantly lower in the CoQ10 group compared to the placebo group, but there were no significant differences in serum insulin and HOMA-IR between the two groups. Moreover, there were no significant changes in glycemic control indices after supplementation with Q10.

**Table 4:** Glycemic control indices before and after intervention

**Variable**	**Before intervention (Mean ± SD)**	**After intervention (Mean ± SD)**	**P-value**
**FPG (mg/dl)**			
Q10 (n = 20)	131.45 ± 36.3	115.95 ± 20.7	NS^(c)^
Placebo (n = 20)	132.75 ± 38.2	152.55 ± 65.2	0.03
P-value	NS^*(a)^	0.02^(b)^	
**Insulin (μm/ml)**			
Q10 (n = 20)	9.23 ± 6.6	8.58 ± 6.6	NS
Placebo (n = 20)	11.02 ± 4.8	10.80 ± 5.5	NS
P-value	NS	NS	
**HOMA-IR**			
Q10 (n = 20)	3.14 ± 2.9	2.59 ± 2.4	NS
Placebo (n = 20)	3.61 ± 1.9	4.44 ± 4.9	NS
P-value	NS	NS	
**HbA**_**1**_**C (%)**			
Q10 (n = 20)	6.83 ± 0.8	6.44 ± 0.9	NS
Placebo (n = 20)	6.95 ± 1.1	7.18 ± 1.1	NS
P-value	NS	0.01	

*NS: Not Significant.

P < 0.05 is considered as significant.

^(a)^Based on independent t-test.

^(b)^Based on ANCOVA adjusted for baseline values.

^(c)^Based on paired t-test.

CoQ10: Coenzyme Q10; FPG: Fasting Plasma Glucose; HOMA-IR: Homeostatic Model Assessment; HbA_1_C: Hemoglobin A_1_C.

## Discussion

The aim of the present study was to investigate the effects of 12 weeks CoQ10 supplementation on the lipid profile and glycemic determinants in patients with diabetes. According to the results of this study LDL-C and total cholesterol were significantly higher in the Q10 group. Although LDL-C significantly increased after 12 weeks of supplementation with Q10, triglyceride and HDL-C decreased after intervention.CoQ10 reduced HDL-C and total cholesterol.

Our findings confirmed the results of some previous studies on rats and humans. Witting et al. demonstrated that Q10 supplementation had no effect on cholesterol concentration in apolipoprotein E gene knockout mice [22]. Kunitomo et al. indicated that 6 weeks Q10 treatment had no effect on dyslipidemia in a rat model of metabolic syndrome [18]. In line with these studies, Chew et al. investigated the effects of fenofibrate and coenzyme Q10 in type 2 diabetic subjects with left ventricular diastolic dysfunction (LVDD). The findings of this study showed that daily consumption of 200 mg Q10 does not affect total cholesterol, HDL-C and LDL-C [20]. These results were similar with the findings of Playford et al. in patients with diabetes [19]. It has been shown that intake of 40 mg/kg Q10 for 4 weeks significantly reduces the serum levels of triglycerides, total cholesterol, LDL-C and Very Low Density Lipoprotein Cholesterol (VLDL-C) and increases HDL-C in streptozotocin induced diabetic rats [16]. In general, human studies do not confirm the lipid lowering effects of Q10 in patients with diabetes [18]-[20],[22].

LDL-C and total cholesterol were significantly higher in the Q10 group compared to the placebo group. Although LDL-C significantly increased after 12 weeks of supplementation with Q10, triglyceride and HDL-C decreased after intervention. There are reports of Q10 supplementation unfavorable effects on lipid profile in certain subgroups of patients such as osteoporotic patients. It is time to ask whether diabetics are among these patients or not?

According to the results of the present study, after intervention FPG and HbA_1_C were significantly lower in the CoQ10 group compared to the placebo group, but there were no significant changes in glycemic control indices after intervention within the CoQ10 group.

These findings were in line with many previous published studies. Coenzyme Q10 has been known as an enhancing glycemic control factor acting through several mechanisms, comprising a decrease in oxidative stress [23],[24]. Modi et al. showed that administration of Q10 in streptozotocin induced diabetic rats leads to decreasing in serum levels of glucose significantly but serum concentrations of insulin was not changed. HbA_1_C was not measured in their study [16]. Sena et al. demonstrated that consumption of Q10 supplement reduces HbA_1_C in diabetic rats [17]. Also, Palyford et al. concluded that CoQ10 significantly decreases HbA1c. Similar with these findings, Hodgson et al. showed that daily intake of 200 mg Q10 in patients with diabetes results in decreasing of HbA1C and consequently long term improvement of glycemic control [19]. Conversely, the results of a number of published studies revealed no difference in glycemic control and requirement of insulin [25]-[27].

It has been shown that, Q10 levels are reduced in patients with diabetes, and might be responsible for *β* cell depressed function in type 2 diabetes [28]. Q10 impaired levels may induce insulin resistance via mitochondrial dysfunction [29].

There is an increased oxidative stress in patients with diabetes. Hyperglycemia increases ROS formation which activates proteins and pathways such as protein kinase C, polyol, nuclear factor-kappaB and protein kinases (JNK/SAPK).

Increased functional proteins glycosylation and glucose auto-oxidation are among other responsible mechanisms for ROS production and resulted in lipid peroxidation [30]. Q10 could enhance fatty acid oxidation through AMPK- mediated (AMP activated protein kinase) peroxisome proliferator-activated receptor α (PRARα) stimulation [31] which in turn increases lipoprotein lipase and APO-AV expression. This may decreases TG an VLDL Levels [32].

Additionally it has been shown that PRARα agonist could increase LDL-C size which is more protective against vascular diseases [33]. PRARα can inhibit fatty acid and TG synthesis via reduction in SREBP1c and SREBP-2 maturation [34].

Antioxidant reserves have been decreased in patients with diabetes. Q10 supplementation may compensate this impaired antioxidant system and decrease oxidative stress in these patients.

Free fatty acids (FFA) and glucose overload in patients with diabetes tend to increase acetyle-COA production, and increase electron donors from TCA cycle and enhance membrane potential. Increased membrane potential improve free radicals intermediates of Q10 half life that reduces O_2_ to superoxide [35].

It has been shown that antioxidant treatment may reduce glucose levels [36] via β cell protection against ROS and glucose toxicity, and increased insulin secretion [35].

According to the results of our study HOMA-IR index and insulin levels did not change significantly, however HbA1c levels were significantly decreased. Due to short half-life of insulin, its level does not reflect its mean level in a prolonged period of time. One of the main strength of this study was measurement of Q10 levels before and after intervention.

There were some limitations in this study. The major limitation was that the inclusion criteria were wide. Also, it would be better if we applied three-day 24- hour recall instead of one-day 24- hour recall to evaluate our patient’s diet.

Proinsulin and C-peptide levels are better markers to show insulin production and β cell function; however these parameters were not measured. Measurement of C-peptide levels would be beneficial, since insulin concentration in the portal vein ranges from two to ten times higher compared to the peripheral circulation. Additionally fasting intact pro insulin could be used as a specific predictor of insulin resistance in type 2 diabetes [37].

Finally, it should keep in mind that uncontrolled and OTC consumption of any supplement could accompany by several unfavorable and hazardous effects [38]. In the case of Q10 supplementation for diabetics, lipid profile impairment needs special care.

## Conclusion

The results of the present study showed that 12 weeks supplementation with Q10 may improve glycemic control without favorable effects on lipid profiles of patients with diabetes. Further larger studies are warranted to confirm these findings.

## Competing interests

The authors declare no conflict of interests.

## Authors' contribution

HZ participated in the statistical analysis, data interpretation and article writing. SE participated in the study design. SS participated in the statistical analysis and data interpretation. NR participated in the study design. FS participated in the study design and data interpretation. IH participated in the data acquisition and interpretation. BG participated in the study design and statistical analysis. SJ participated in the study design, data interpretation, statistical analysis and article writing. All authors read and approved the final manuscript.
